# Patient and Family Engagement in the Design of a Mobile Health Solution for Pediatric Asthma: Development and Feasibility Study

**DOI:** 10.2196/mhealth.8849

**Published:** 2018-03-22

**Authors:** Andrew McWilliams, Kelly Reeves, Lindsay Shade, Elizabeth Burton, Hazel Tapp, Cheryl Courtlandt, Andrew Gunter, Michael F Dulin

**Affiliations:** ^1^ Center for Outcomes Research and Evaluation Carolinas HealthCare System Charlotte, NC United States; ^2^ Department of Family Medicine Carolinas HealthCare System Charlotte, NC United States; ^3^ Community Care Partners of Greater Mecklenburg Carolinas HealthCare System Charlotte, NC United States; ^4^ Department of Pediatrics Carolinas HealthCare System Charlotte, NC United States; ^5^ Academy of Population Health Innovation Department of Public Health Sciences University of North Carolina Charlotte Charlotte, NC United States

**Keywords:** engagement, pediatric asthma, shared decision-making, health information technology

## Abstract

**Background:**

Asthma is a highly prevalent, chronic disease with significant morbidity, cost, and disparities in health outcomes. While adherence to asthma treatment guidelines can improve symptoms and decrease exacerbations, most patients receive care that is not guideline-based. New approaches that incorporate shared decision-making (SDM) and health information technology (IT) are needed to positively impact asthma management. Despite the promise of health IT to improve efficiency and outcomes in health care, new IT solutions frequently suffer from a lack of widespread adoption and do not achieve desired results, as a consequence of not involving end-users in design.

**Objective:**

To describe a case study of a pediatric asthma SDM health IT solution’s development and demonstrate a methodology for engaging actual patients and families in IT development. Perspectives are shared from the vantage point of the research team and a parent of a child with asthma, who participated on the development team.

**Methods:**

We adapted user-centric design principles to engage actual users across three main development phases: project initiation, ideation, and usability testing. To facilitate the necessary level of user engagement, our approach included: (1) a Development Workgroup consisting of patients, caregivers, and providers who met regularly with the research team; and (2) “real-world users” consisting of patients, caregivers, and providers recruited from a variety of care locations, including safety-net clinics.

**Results:**

Using this methodology, we successful partnered with asthma patients and families to create an interactive, digital solution called Carolinas Asthma Coach. Carolinas Asthma Coach incorporates SDM principles to elicit patient information, including goals and preferences, and provides health-literate, tailored education with specific guideline-based recommendations for patients and their providers. Of the patients, caregivers, and providers surveyed, 100% (n=60) said they would recommend Carolinas Asthma Coach to a friend or colleague. Qualitative feedback from users provided support for the usability and engaging nature of the app.

**Conclusions:**

This project demonstrates the feasibility and benefits of deploying user-centric design methods that engage real patients and caregivers throughout the health IT design process.

## Introduction

Asthma is a highly prevalent, chronic disease with significant morbidity, cost, and disparities in outcomes [1-5]. Despite the availability of effective treatment options, many patients with asthma lack adequate symptom control and almost 50% have symptoms more than once per week [6,7]. Improving patient engagement, self-management, and provider adherence to guideline-based therapy may help improve asthma symptoms [8-10]. One modality associated with improved patient engagement and asthma outcomes is shared decision-making (SDM), which is a process whereby patients and clinicians work together to incorporate evidence, preferences, and values into treatment decisions. Widespread adoption of SDM into practices is challenged by staffing shortages (eg, limited personnel who can assume a health coaching role), staff turnover, and provider time constraints in volume-based reimbursement models [11-13]. These challenges of integrating SDM into everyday practice, as well as personalizing complex asthma guidelines, can both be addressed by leveraging health information technology (IT) applications [14].

A health IT app that enables SDM for pediatric asthma must uniquely deliver an experience that is useful to all end-users: caregivers, patients, and providers. Furthermore, to ensure that an app addresses asthma disparities, it must be designed to be accessible and understandable by populations who have limited health literacy [15,16]. Unfortunately, health IT apps frequently do not achieve desired results as a consequence of inadequately involving this full spectrum of end-users in their designs [16-18]. This absence of end-user involvement is particularly prevalent for those patients with additional barriers to accessing quality medical care, such as the underserved and chronically ill; however, these groups may stand to gain the most from health improvements offered by emerging health IT solutions [19]. Indeed, for health IT to be successful, end-user alignment must begin at project inception by first understanding who the users are, then asking them what they want and need, followed by ongoing testing of a solution’s usability and responsiveness to addressing identified needs [20-22].

While there is growing recognition of the need for this level of user engagement in design, there are limited studies demonstrating methods of how to achieve this in health care settings. Moreover, despite the opportunity for health IT to alter the trajectory of health disparities, there is a paucity of research on understanding best practices for engaging underserved patients in the design and implementation of health IT interventions [23].

As we set out to create a digital app for pediatric asthma SDM, we aimed to develop a design process that truly engaged the diverse cast of users involved in caring for a pediatric asthma patient. The interactive digital solution, called Carolinas Asthma Coach, incorporates SDM principles to elicit patient information (including goals and preferences) and provides health-literate, tailored education with specific guideline-based recommendations for patients and their providers. Here we describe the approach used to engage pediatric patients, their caregivers, and their providers, while providing additional perspectives from Beth, a parent who participated on the development team.

## Methods

### User-Centric Design Approach

**Figure 1:**
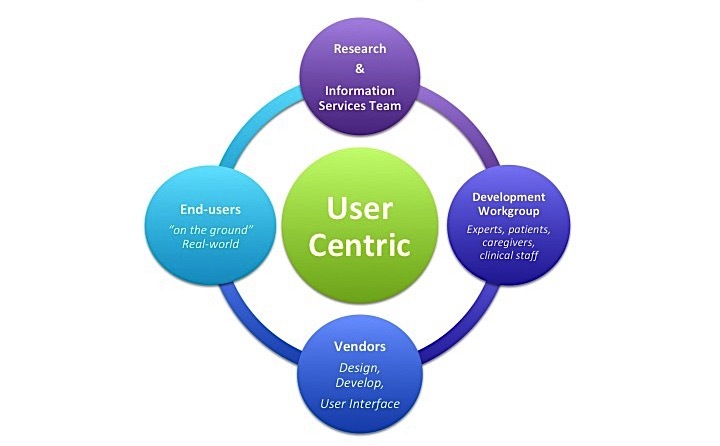
User-centric design process to engage the Development Workgroup and real-world end-users.

We created a process that partnered researchers, IT experts, patients, caregivers, and providers to develop patient- and provider-centered health IT solutions. This approach incorporates user-centric design principles to collaborate with end-users throughout a health IT project’s ideation, design, pilot testing, implementation, and evaluation phases (Figure 1). To facilitate this level of engagement and ensure broad representation, we created a Development Workgroup that met regularly with the research team and consisted of seven representative patients, caregivers, and providers who were involved in pediatric asthma care (and who were recruited through existing contacts with the research team). Whenever substantive changes were made to the solution, the research team solicited feedback and approval from this Development Workgroup. Further testing was then performed with “real-world users” consisting of pediatric asthma patients aged 7 to 17 years, their caregivers, and their providers, who were recruited from the health care system’s primary care clinics, the Children’s Emergency Department, and a Children’s Hospital located in Charlotte, North Carolina. A research coordinator located in these various clinical settings used convenience sampling to recruit pediatric patients and their caregivers as they presented for visits related to asthma. To ensure that we adequately addressed health literacy, technical literacy, and social contexts, we intentionally performed preliminary testing work within safety-net clinics.

When developing the Carolinas Asthma Coach, our approach to user-centric design included three phases: *Initiation*, *Ideation*, and *Usability*. First, in the *Initiation Phase*, we gathered information to help understand potential users’ needs and barriers using: (1) key informant interviews with providers and caregivers selected by convenience sampling, and (2) reviewed focus group results from previous asthma research conducted in local clinics [24]. This phase was followed by the *Ideation Phase* **,** in which we engaged the Development Workgroup to conceptualize a virtual tool and possible workflows for asthma SDM [25]. Finally, the project entered the *Usability Phase*, in which we conducted real-world user testing. To allow for iterative development, we solicited this real-world feedback at three distinct time points. First, at the paper prototype stage of the solution, we vetted the script content for tone and meaning with a health literacy consultant, patients, caregivers, and providers. With each iteration, we revised the scripting based on feedback. Second, in the preliminary production stage, we solicited feedback on all critical segments, which included rough cuts combining scripts, illustrations, and animations.

**Figure 2:**
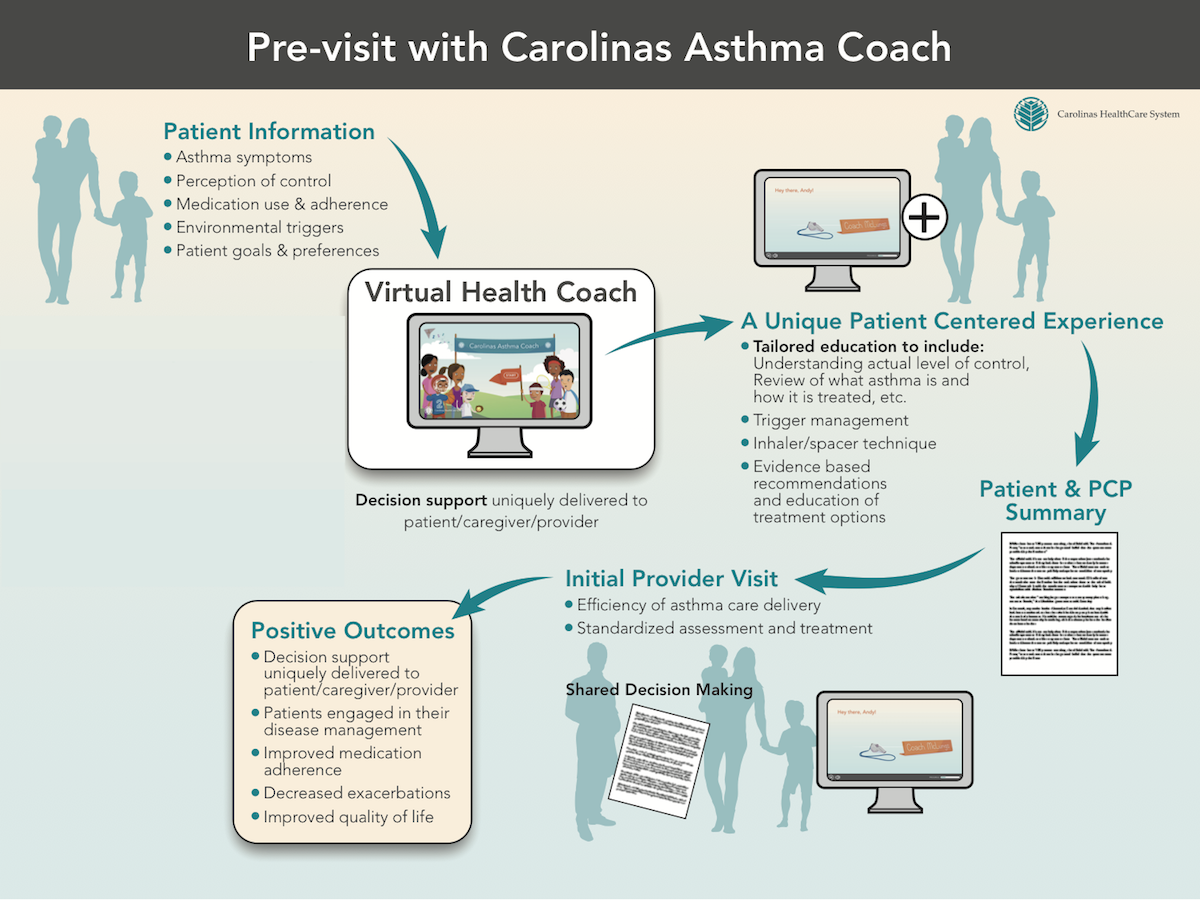
Process diagram for use of Carolinas Asthma Coach in a Primary Care Provider (PCP) visit setting.

Third, with the fully-produced version of the product, we conducted quality assurance testing, while soliciting feedback on both the overall experience and the solutions’ summary pages. Furthermore, at this stage we observed patients and caregivers as they interacted with the Asthma Coach prior to an asthma visit with a primary care provider. Following the visit, we conducted in-depth interviews regarding the experience and asked structured questions about satisfaction and likelihood of recommending the intervention.

### Intervention Description

Carolinas Asthma Coach is an interactive health IT-enabled solution designed to facilitate SDM, encourage self-management, and drive standardized, evidence-based care. The Asthma Coach is a Web-based app built on a platform that incorporates branching technology to navigate through the HTML5 multi-media experience, which emulates the key humanistic components of in-person health coaching (see Multimedia Appendix 1 for additional information and screenshots). The app incorporates elements of SDM by using a conversational style to: (1) elicit patient information (symptoms, perception of asthma severity or control, medication adherence, triggers, and goals); (2) provide tailored education (asthma background basics, inhaler technique, trigger avoidance); and (3) incorporate motivational interviewing techniques. Additionally, clinical decision support is woven into the conversation through background analytics and logic that allow the Asthma Coach to determine asthma disease severity or control and recommend treatment options, which are individually filtered from up-to-date evidence. Designed to be completed prior to an asthma-specific provider visit, the Asthma Coach sets the stage for SDM, where patients and caregivers are better informed, resulting in a more meaningful and efficient visit with their providers (Figure 2).

## Results

### Reflection From a Workgroup Participant

The following is a reflection from a parent, Beth, who was a participant in the development workgroup:

I am a busy working mother of four children ages 7-23, three of whom have asthma. The three that have asthma are not at all the same. Each child is developmentally different; they are on different medicines, and the things that trigger their asthma are unique. It is difficult to keep all their medications straight and monitor their asthma, but I know each of them, and I know what works well, at least most of the time. I worry about each of them as any mother worries. As they grow and develop, my hope and goal is for them to be able to manage their asthma independently, as they are able. How do I teach them to be advocates for their health? I feel that I can recognize a good pediatrician when he or she walks into the exam room. A good pediatrician talks with my child and me and does not talk at us. When pediatricians ask our opinions, our beliefs, our concerns, and our goals, I know they are going to work with me. I believe that this shared team approach is critical to deciding on the best treatment plan. When I was approached about working on Carolinas Asthma Coach, I was excited about the opportunity to partner and help create a tool that might benefit all children with asthma and will assist parents to become active partners in our children’s care.

As a parent stakeholder in the design of Carolinas Asthma Coach, I met with the research team often to give my opinions on content, flow, and the approach of the app. I was not only listened to, but my feedback was also incorporated into future iterations of the Asthma Coach. My 16-year-old daughter was asked to test the app. She had been self-sufficient in taking her medication and independently monitoring her asthma. As I watched her answer the questions, I was startled by some of her responses. I thought her asthma was doing well, but as it turns out she had actually been struggling… unable to sleep well or participate fully on her swim team! Her asthma was not well controlled, as I had assumed! Carolinas Asthma Coach’s results prompted me to make an appointment with the doctor. She shared with her doctor her challenges and her goal of doing better on the team. We decided together to change her medication. This improved her control and ultimately her endurance on the swim team.

### Example Feedback From Real-World End-Users in the Usability Phase

In the preliminary production stage, examples of user feedback on animations included: (1) the animation of the airways during an exacerbation did not convey the intended depiction of inflammation; (2) animations of children should be doing physical activity, rather than using electronics; (3) an initial theme of mountain climbing did not resonate well with children, who instead suggested a sports theme; and (4) an animated character demonstrating an exacerbation appeared to be in too much distress, invoking a feeling of fear in the user. With each of these examples, improvements were made based on feedback and then tested again with users and the Development Workgroup.

An example of user feedback and resultant changes during the piloting of the fully-produced version of the Carolinas Asthma Coach was that both providers and caregivers reflected that the summary pages were too long and critical information needed more emphasis. Based on this feedback, the summary content was shortened to a concise single page and yellow highlighting was added for critical elements.

When patients, caregivers, and providers were surveyed while testing the fully-produced version, 100% (n=60) said they would recommend Carolinas Asthma Coach to a friend or colleague. Additional comments from patients, caregivers, and providers about their experiences using Carolinas Asthma Coach in the clinic prior to an asthma visit included:

I didn’t understand what asthma was before… this (Carolinas Asthma Coach) made it easy to understand.

Parent

I need to get a spacer because the Asthma Coach said it was important.

Parent

It (Carolinas Asthma Coach) is friendly and funny.

Pediatric patient

It helped me to know what questions to ask the doctor.

Pediatric patient

When we can do this (implement the standardization and efficiency of Carolinas Asthma Coach) it is going to be really big for asthma care.

Provider

## Discussion

### Prinicipal Findings

By leveraging the combination of a Development Workgroup and frequent usability testing in real-world clinics, we successfully engaged end-users in the development of a health IT app for pediatric asthma SDM. The approach presented here offers an example of how to incorporate user-centric design methods with an intentional focus on inclusion of vulnerable populations. Regardless of the approach used, these results highlight that it is critical to specifically engage with and address the needs of patients, caregivers, and providers throughout the health IT design process.

While our approach to end-user engagement helped to ensure that we produced a useful product, it was not without challenges. Perhaps the biggest challenge was that this level of engagement and iteration slowed our development down by approximately six months. Efficiency might be improved if more resources were applied to the project; for example, to allow simultaneous patient recruitment at multiple sites.

Second, recruitment of patients and caregivers within real-world clinic settings proved difficult. Issues included: the time commitment involved in usability testing for participants, not disrupting workflows in busy clinical environments, space limitations for testing, and patients who were prescreened but did not show up for appointments. Despite these challenges, the pay-off in terms of the improved usability from this level of engagement was well worth the additional effort and expense.

The following is Beth's thoughts on her role in the development of Carolinas Asthma Coach:

As health care evolves, health IT solutions can help patients and doctors better connect. My role in the development of Carolinas Asthma Coach, I think, helps to demonstrate how these tools can be that much more helpful, when a parent and patient have a hand in creating the solution they will use. My recommendation for health IT is to ensure that the voice of the user is included every step of the way.

### Conclusion

This project demonstrates the feasibility and benefits of deploying user-centric design methods that engage real patients and caregivers throughout the health IT design process. Furthermore, Carolinas Asthma Coach provides an example of how this approach can produce a solution that is acceptable and useful for patients, caregivers, and providers.

## Appendix

Multimedia Appendix 1 Shared decision making intervention description: Carolinas Asthma Coach.

## Abbreviations

IT: information technology
SDM: shared decision-making
