# Supplementary material for: Patient and Family Engagement in the Design of a Mobile Health Solution for Pediatric Asthma: Development and Feasibility Study
Source: JMIR Mhealth Uhealth. 2018 Mar 22;6(3):e68. doi: 10.2196/mhealth.8849 (PMC5887041; doi:10.2196/mhealth.8849)
Supplement: Multimedia Appendix 1 [file mhealth_v6i3e68_app1.pdf]

Appendix 1: Shared decision making Intervention description: Carolinas Asthma Coach

Based on the platform of motivational interviewing and shared decision making, the ipad version of the Asthma Coach was designed to facilitate shared decision making, encourage self-management, and drive standardized, evidence-based care. The tool is interactive, designed with branching structure, so as to provide and gather tailored information that guides the negotiation/ deliberation step between patient and provider. During the iterative development we partnered with researchers, IT experts, a health literacy expert (Janice Scotton, a patient experience consultant at Carolinas Healthcare with health literacy expertise) patients, caregivers, providers, and stakeholders to create an interactive, digital health coaching experience for pediatric asthma (demo at https://chsac.jellyvision-conversation.com/staging/html5) The coach incorporated patient- and user- centered design principals throughout the design, pilot testing, implementation, and evaluation phases with the goal of developing an asthma shared decision making tool.

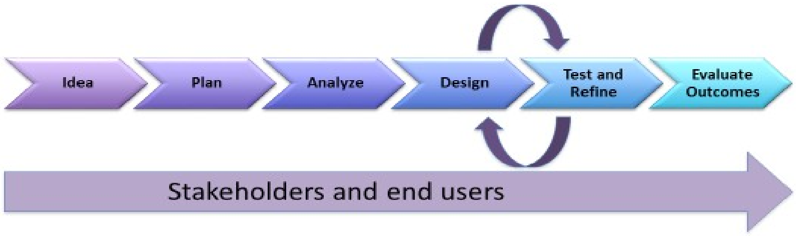

How does it work?

Asthma Coach is designed to facilitate shared decision making, encourage self-management, and drive standardized, evidence-based care. Illustrated in the table below, 8 year old Kayla presents to the Emergency Room with an acute asthma exacerbation. Before she meets with her doctor and while she receives an albuterol nebulizer treatment, Kayla and her Mom engage with Carolinas Asthma Coach. The coach relates to Kayla early by asking “How are you feeling about your asthma visit?” and by using humor “I’m here to coach you how to Kick Asthma right square in the butt!”. Humor eases anxiety to participate in SDM.

During this coaching session, the Coach gathers information about her goals, adherence, symptoms, preferences and provides tailored education about asthma, triggers, and treatments. At the end of the coaching session, personalized summary pages are generated for both the patient and provider. With the aid of the summary, the patient, caregiver and provider share in the decision-making and create a personalized treatment plan at the point of care.

**Introduction**	**Carolinas Asthma Coach “Start” Screen.**	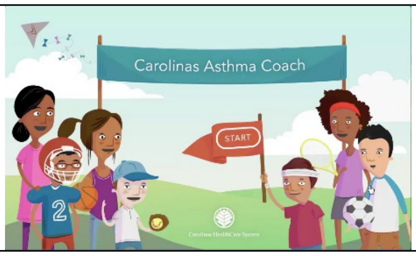

<table>
<tr>
<td>Goal Setting</td>
<td>What's the one thing asthma makes super hard for you? (GOAL)

Staying Active (like playing sports or just running around)
Sleeping through the night
Going to school
My asthma not that bad
Something else
</td>
<td>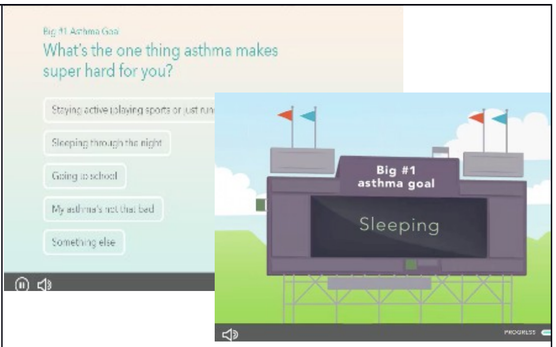
</td>
</tr>
<tr>
<td>Information Exchange</td>
<td>Included Educational Videos:

Asthma Action Plan
Symptoms of Asthma
What is asthma?
Difference between controller and rescue
Inhaler technique
Trigger avoidance strategies
</td>
<td>Examples: What is asthma?
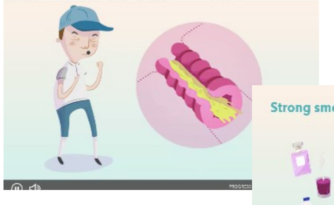
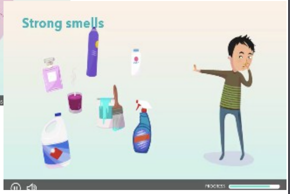

Trigger Avoidance</td>
</tr>
<tr>
<td>Asthma Control Questions</td>
<td>Which of these symptoms do you get?

Tight chest
Coughing
Wheezing
Hard to breathe
</td>
<td>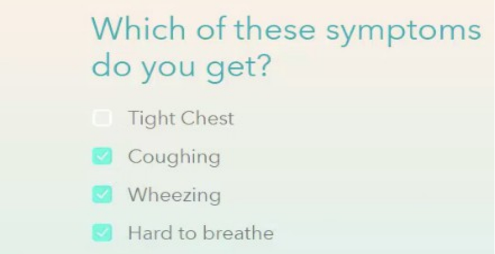
</td>
</tr>
<tr>
<td></td>
<td>Do you take a Controller medicine? (like Qvar, Flovent, Advair, Pulmicort)

Yes
No
</td>
<td>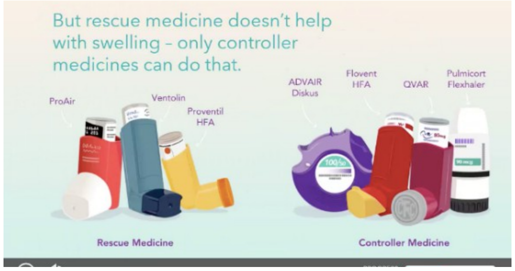
</td>
</tr>
<tr>
<td></td>
<td>How many days a week do you use your controller medicine?

One or two
Three or four
Five or six
Everyday
Never
</td>
<td>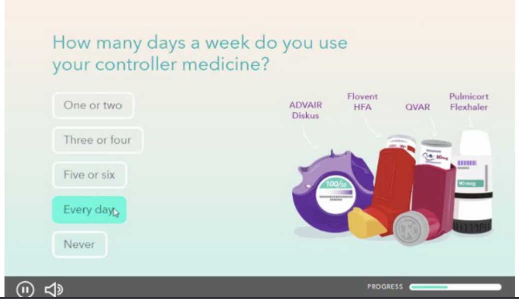
</td>
</tr>
</table>

	**Now think about your asthma lately, does it feel like it's under control?**<br>• Yes!<br>• Kind of!<br>• Nope!	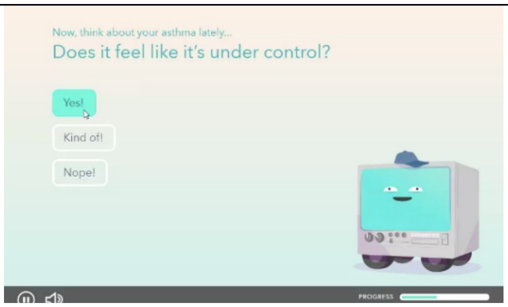
	**Think back over the last month, how many days each week have you had asthma symptoms during the daytime?**<br>• Never<br>• 1 or 2 days each week<br>• 3 or 7 days each week<br>• Many times a day each week	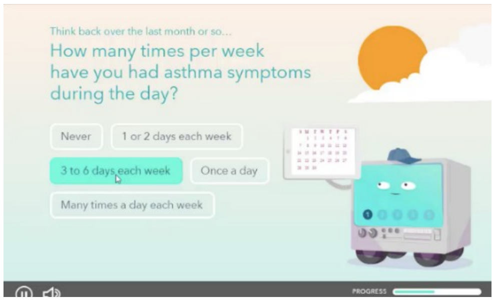
	**In the last month, how many nights did asthma symptoms wake you up?**<br>• Never<br>• 1 night<br>• 2 or more nights total<br>• 2 or more nights each week	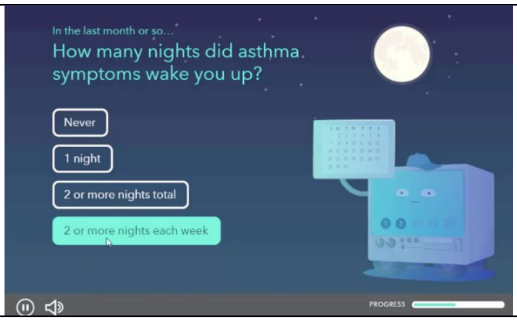
	**In the last month, how often have you had to use a rescue inhaler or nebulizer- day or night-because you were having trouble breathing?** That inhaler might have a weird name like albuterol, ProAir, Ventolin, Xopenex. (don't count those times when you used your inhaler before exercise)<br>• Never<br>• 1 or 2 times a week<br>• 3 to 7 times a week<br>• Several times a day<br>• What are those?<br>• I don't have one of those	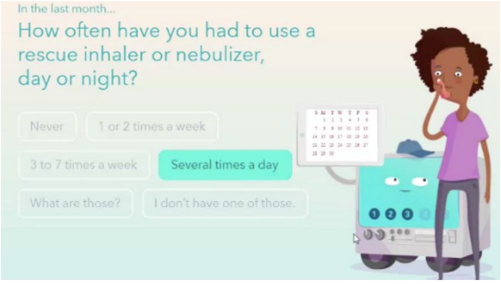
	**In the last month, how easy has it been for you to run and play without asthma getting in the way?**<br>• Super easy<br>• Kind of easy<br>• Not very easy	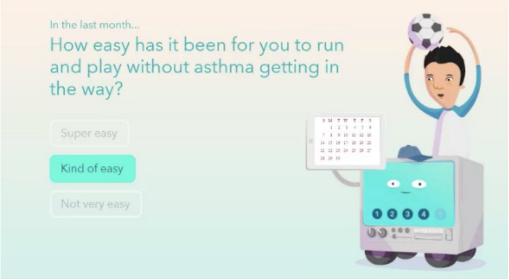

	**In the last year, how many asthma attacks did you have that made you take oral steroids (like Prednisolone, Prednisone, Orapred, Prelone)?**<br>• None<br>• Just 1<br>• 2 or 3<br>• 4 or more	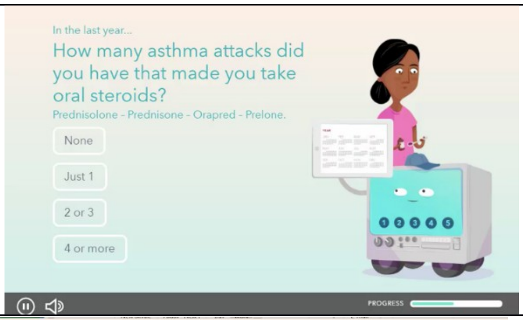
**Asthma Triggers Specific to Patient**	**Which of these triggers make your asthma worse: (Check all that apply)**<br>Exercise Pets<br>Mold Dust<br>Smells Smoke Pests<br>Getting really happy, sad. or angry<br>Getting sick (like having a cold)<br>Changes in the weather<br>Pests<br>Other?	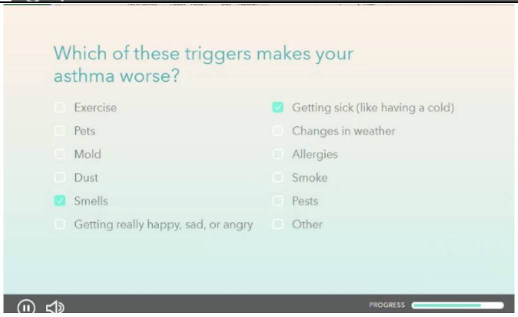
**Risk Questions:**	**Has (name) ever been admitted to the intensive care unit (ICU) for asthma?**<br>• Yes<br>• No<br><br>**Has (name) ever been intubated (breathing tube) for asthma?**<br>• Yes<br>• No	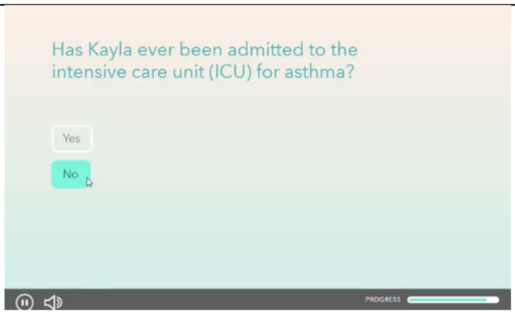
	**In the last 12 months, has (name) had asthma care in the emergency room (ER) or urgent care?**<br>• Yes<br>• No<br><br>**In the last 12 months, has (name) had to stay overnight in the hospital because of asthma?**<br>• Yes<br>• No	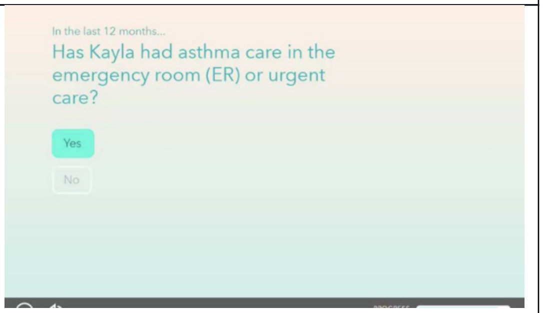
**Comparison of Patient's Perception of Control vs. Actual Level of Control**	**Control-O- Meter**	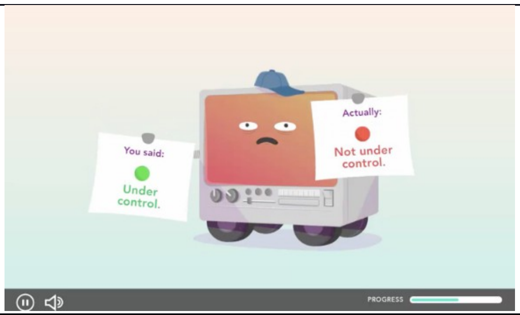

<table>
<tr>
<td>Concerns and Preferences for Treatment Choices:</td>
<td>
Pick all that apply…when it comes to asthma medicine, what concerns do you have?

Will it control the asthma?
How much will it cost?
What are the side effects?
How hard is the medicine to use and how often do I use it?

</td>
<td>
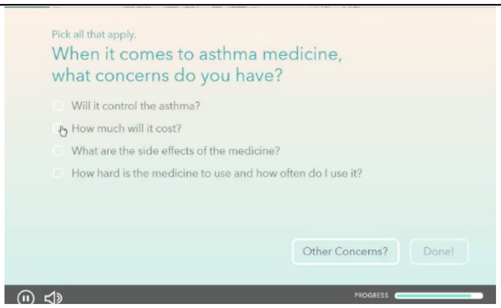

</td>
</tr>
<tr>
<td>Evidence-based Step Wise Treatment Options</td>
<td colspan="2">
Medication Options based on NHLBI 2007 guidelines

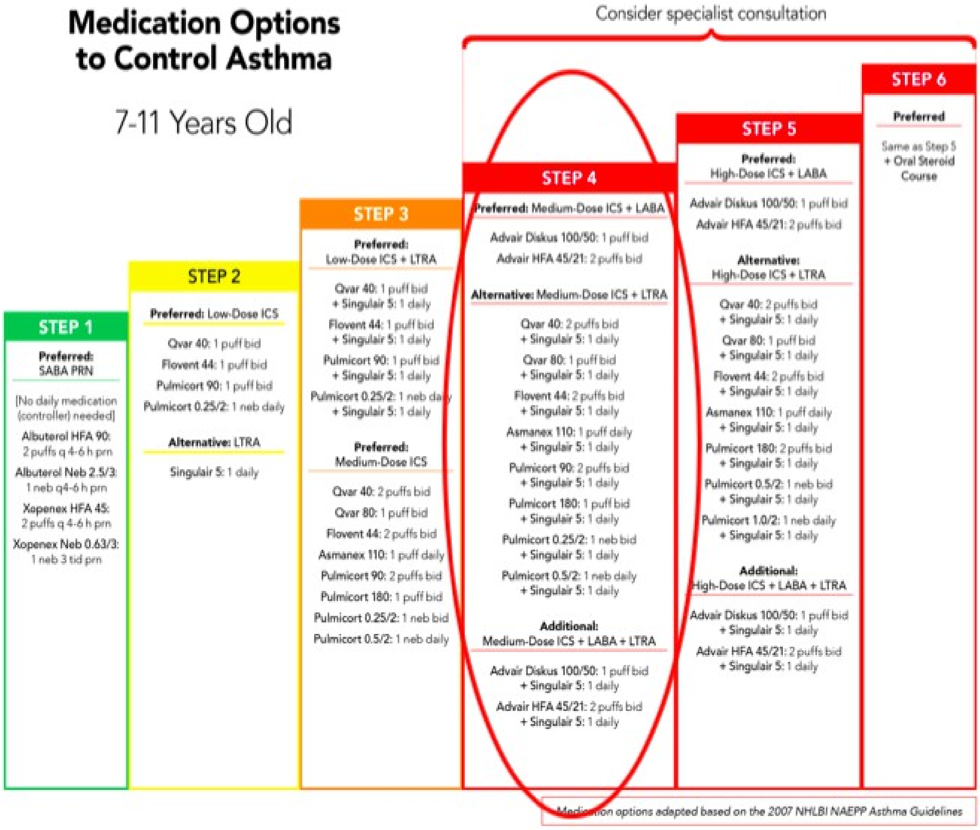

</td>
</tr>
</table>

Patient Summary Handout:

The Playbook summaries the conversation with Coach McLungs:

- Starts with key points to discuss with the doctor
- Lists Kayla's preferences and concerns
- Lists Kayla's goal
- Lists Kayla's perception of control as compared to her actual level of control
- Review of education content discussed

Kayla's Asthma Visit Playbook

Hi, Kayla!

This playbook has what you need to make your asthma visit go well. It can also help you with helpful facts about your asthma at home!

Key Points to Discuss with Provider

Mom, talk to the doctor about:

- Staying on the medicine or considering a change in the amount taken
- When to schedule a follow-up appointment
- Your asthma medicine concerns:
  - Control
  - Convenience
  - Side effects
- Your other asthma questions:
  - Can Kayla play basketball?

Preferences/Concerns to be Considered During Shared Decision Making

Patient-centered Goal

Kayla's Big #1 Asthma Goal

You said you wanted to stay active. Controlling your asthma will help reach that goal!

What is Asthma, Again?

Asthma is a "chronic" condition–meaning it won't just go away. You need to control it with the right medicine and smart choices.

Kayla's Asthma Symptoms

Here are the symptoms you told me you get:

- Tight Chest
- Coughing

Be aware of your symptoms! Your Asthma Action Plan will help you know what to do when you start to feel them.

Kayla's Asthma Action Plan

Your doctor will help you make an Asthma Action Plan. With an Asthma Action Plan, you'll know what to do if your asthma symptoms are good, okay, or bad. Like this:

Asthma Education Review

Green Zone: No asthma symptoms. Just keep taking your daily controller medicine, if ordered by your doctor.

Yellow Zone: Some asthma symptoms. Be careful! Keep taking your controller medicine and add your rescue medication as the doctor ordered.

Red: Lots of asthma symptoms! Take your rescue medication as ordered and **Get help from a grown-up quick!**

Kayla's Control-O-Meter

You take a controller medicine almost every day. That's pretty good, but you should be taking it every day.

Patient's Perception of Control vs. Actual Level of Control

You thought your asthma was kind of under control. But according to the Control-O-Meter… your asthma is persistent asthma, very poorly controlled. But you can make it better! That is why you need to talk with your doctor.

You said you were having problems with:

- Asthma symptoms during the day
- Asthma symptoms waking you up at night
- Needing to take rescue medicine
- Running and playing

How do you **make it better?** By using your medicine and following your asthma action plan, of course!

How to Use an Inhaler:

- Make sure you have your spacer.
- Shake the inhaler.
- Put the spacer on the inhaler.
- Put the spacer in your mouth.
- Press down on the inhaler and breathe in, nice and slow…
- Hold your breath for 10 seconds then breathe out slowly.
- Wait 1 minute–count to 60 if you have to!
- If your doctor tells you to take two puffs, do it all over again!

Asthma Educ[illegible]
Review

Controller Medicine or Rescue Medicine?

Controller medicine: It may have a funny name like Qvar, Pulmicort, Flovent, or Advair. Take it everyday before your asthma symptoms have a chance to bother you. It will stop the breathing tubes in your lungs from swelling up and making too much mucus.

Rescue medicine: It might be called albuterol, ProAir, Proventil, Ventolin, or Xopenex. It should be taken when your asthma symptoms start, like in the yellow zone. Rescue medicine makes the muscles around your breathing tubes relax. That makes it easier to get air through—but that only lasts a little while.

Kayla's Asthma Triggers

Triggers are the things that make your asthma worse. Here are some tips for staying away from triggers:

- Avoid pollution, stay inside on bad air days, and never let anyone smoke near you. It's okay to be kind of bossy about it!
- Just in case you're allergic to cockroaches or mice make sure you always clean up crumbs and never take food into the bedroom.

You said you also had trouble with:

- **Dust:** Look, maybe chores aren't fun, but they can actually help your asthma. Wash your bedsheets, pillowcases, and mattress covers once a week. Vacuum the carpet and furniture in your house once a week, too. Trust me, it'll get rid of a lot of the dust that you can't even see! And if you can, try to stay away from stuffed animals—they tend to get covered in dust really easily.
- **Getting sick (like having a cold):** It's not rocket science: wash your hands, don't be around people with colds. And definitely make sure to get a flu shot every year! Flu + asthma = yikesville.

That's just about it! On the next page, you'll see a colorful chart describing some medication options. Take it to your doctor and ask them to explain how to use it to control your asthma! **Good luck, Kayla!**

—Coach McLungs

The information and materials contained on or in this product do not provide, and are not a replacement for, professional medical evaluation, advice, diagnosis, or treatment. Please see your physician or other health care provider prior to making any decision, or undertaking any action or not undertaking any action related to any health care problem or issue that you may have at any time, now or in the future. Carolinas HealthCare System shall not be liable to you for your reliance on any information obtained through the use of this site, and we do not claim any liability in connection with such information and any services that you might receive from any health care provider through the web site, or based on information on or in the web site. Carolinas HealthCare System makes no warranties, express or implied, regarding the accuracy, completeness, timeliness, comparative nature, or usefulness of any information contained or referenced on the website.

Provider Summary Handout:

Kayla's Asthma Coach results summaries:

- Patient's perception of control
- Patient's goal and preference/concerns
- Reported symptoms
- Identified asthma triggers
- Medication adherence
- Symptom assessment
- Risk factors
- And provides decision support for evidence-based treatment options to negotiate with patient/caregiver
